# A new species of the genus *Phaenochilus* Weise from China (Coleoptera, Coccinellidae, Chilocorini)

**DOI:** 10.3897/zookeys.644.9825

**Published:** 2017-01-10

**Authors:** Wenjing Li, Lizhi Huo, Xiaosheng Chen, Shunxiang Ren, Xingmin Wang

**Affiliations:** 1 Key Laboratory of Bio-Pesticide Innovation and Application, Engineering Technology Research Center of Agricultural Pest Biocontrol, Guangdong Province; Department of Entomology, South China Agricultural University, Guangzhou 510640, China; 2 College of Forestry and Landscape Architecture, South China Agricultural University, Guangzhou 510640, China

**Keywords:** Coleoptera, new species, Phaenochilus, taxonomy, Yunnan

## Abstract

A new species *Phaenochilus albomarginalis* Li & Wang, **sp. n.** is described. The only other species recorded from China is *Phaenochilus metasternalis* Miyatake, 1970 and it is described here for comparison. Diagnoses, detailed descriptions, illustrations, and distributions are provided.

## Introduction

Chilocorini is a medium-sized tribe in the Coccinellidae which was placed in the superfamily Coccinelloidea by recent molecular phylogenetic research on Cucujoidea (Robertson et al. 2015). It consists of 26 genera and about 280 species (Łączyński and Tomaszewska 2012). Chilocorini have been shown to be a monophyletic group by some phylogeny works, but the generic relationships within this tribe are ambiguous (Giorgi et al. 2009; Magro et al. 2010; Seago et al. 2011).

*Phaenochilus* Weise is a small genus of the tribe Chilocorini, the species of which mainly feed on scale insects and a few species of whitefly nymphs. The genus *Phaenochilus* was proposed by Weise (1895). Korschefsky (1932) designated *Phaenochilus punctifrons* as the type species. Giorgi and Vandenberg (2012) revised the genus and described a new species. So far, there are nine species known, distributed mainly in Southeast Asia, China, India and Japan (Giorgi and Vandenberg 2012). Except for *Phaenochilus metasternalis*, which is widely distributed in China and Southeast Asia, the other species of *Phaenochilus* have more restricted distributions.

The genus was unknown from China until Miyatake (1970) described *Phaenochilus metasternalis*. Pang and Mao (1979), Cao et al. (1992), and Ren et al. (2009) redescribed this species, but no new species from China have been added to this genus in recent decades.

In this paper, a second species of *Phaenochilus* from China is described and compared with *Phaenochilus metasternalis*.

## Material and methods

Type specimens of the new species are deposited at the Department of Entomology, South China Agriculture University, Guangzhou, China (SCAU).

External morphological characters were observed with a dissecting stereoscope (SteREO Discovery V20). The following measurements were made with an ocular micrometer:

TL
 total length, length from apical margin of clypeus to apex of elytra 


TW
 total width, width across both elytra at widest point 


TH
 height measured across the highest point of the elytra 


HW
 head width in frontal view 


PL
 pronotal length, from middle of anterior margin to base of pronotum 


PW
 pronotal width at widest point 


EL
 elytral length, from the apex of the elytra to the base including the scutellum 


EW
 elytral width, equal TW 


Male and female genitalia were dissected, cleared in 10% NaOH by boiling for several minutes, and examined with an Olympus BX51 microscope. Genitalic morphological character photographs were generated with digital cameras (AxioCam HRc and Coolsnap-Procf & CRI Micro*Color), attached to the microscopes using AxioVision Rel. 4.8 and Image-Pro Plus 6.0 to capture images from both cameras, and photographs were cleaned up and laid out in plates in Adobe Photoshop CS 8.0.

Morphological terms of Coccinellidae follow Ślipiński (2007) and Ślipiński and Tomaszewska (2010).

## Taxonomy

### Phaenochilus

Taxon classificationAnimaliaColeopteraCoccinellidae

Weise, 1895

Phaenochilus Weise, 1895: 135. Type species: Phaenochilus punctifrons Weise, 1895, by subsequent designation of Korschefsky (1932).

#### Diagnosis.

**Figure 1.**
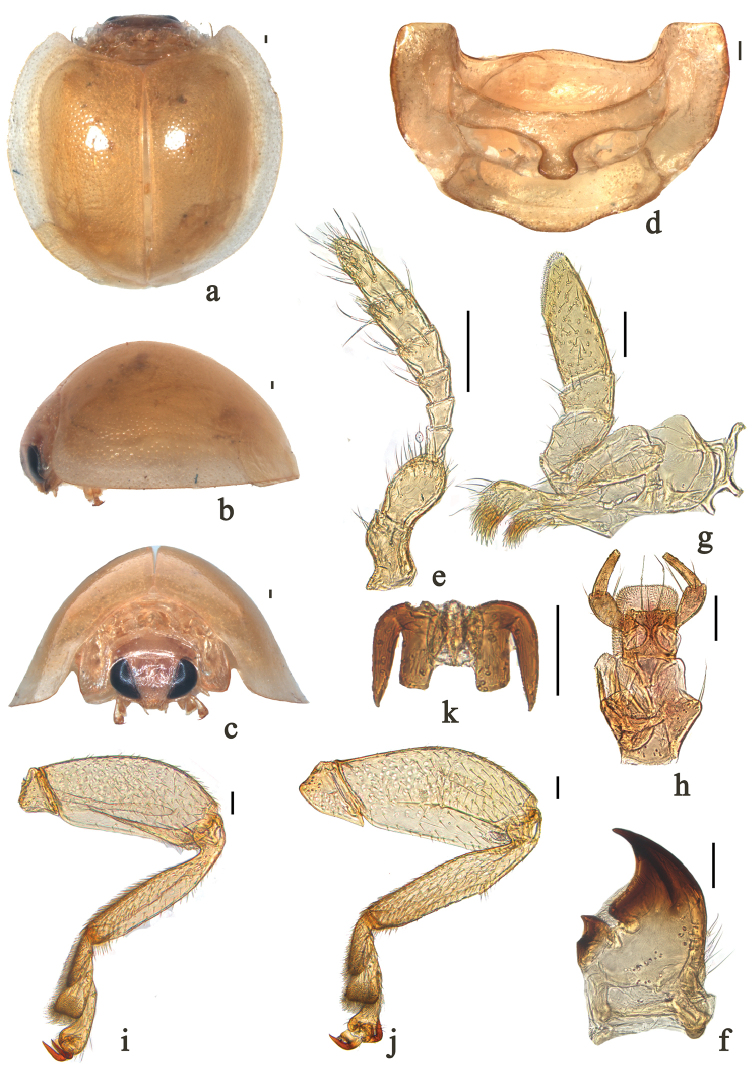
*Phaenochilus albomarginalis* sp. n. **a** dorsal view **b** lateral view **c** frontal view **d** prothorax, ventral **e** antenna **f** mandible **g** maxilla **h** labium **i** front leg **j** hind leg **k** tarsal claws. Scale bars: 0.1 mm.

Members of this genus can be distinguished from other genera of Chilocorini by the following combination of characters: antennae 8-segmented (Fig. 1e); outer margin of mandible slightly curved (Fig. 1f); terminal maxillary palpomere slender and elongate, approximately three times as long as basal width, with sides nearly parallel (Fig. 1g); terminal labial palpomere slender and acuminate, rounded at apex (Fig. 1h); legs without tibial spurs (Fig. 1i–j), tarsal claw stout, with large, rectangular basal tooth about 1/2–2/3 as long as claw (Fig. 1k).

### Phaenochilus albomarginalis

Taxon classificationAnimaliaColeopteraCoccinellidae

Li & Wang sp. n.

http://zoobank.org/DF8F2E9A-2127-43F5-897C-445C9552F76F

**Figure 2.**
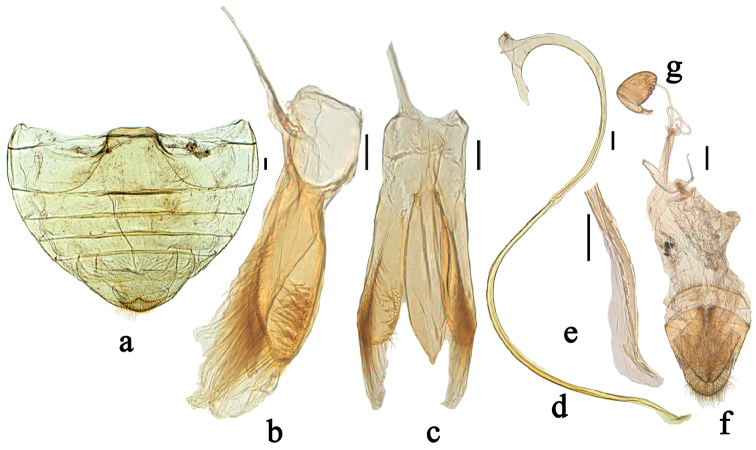
*Phaenochilus albomarginalis* sp. n. **a** abdomen, male, ventral **b** tegmen, lateral view **c** tegmen, ventral view **d** penis **e** apex of penis **f–g** female genitalia: **f** ovipositor **g** spermatheca. Scale bars: 0.1 mm.

Figs 1a–k , 2a–g , 4

#### Diagnosis.

**Figure 3.**
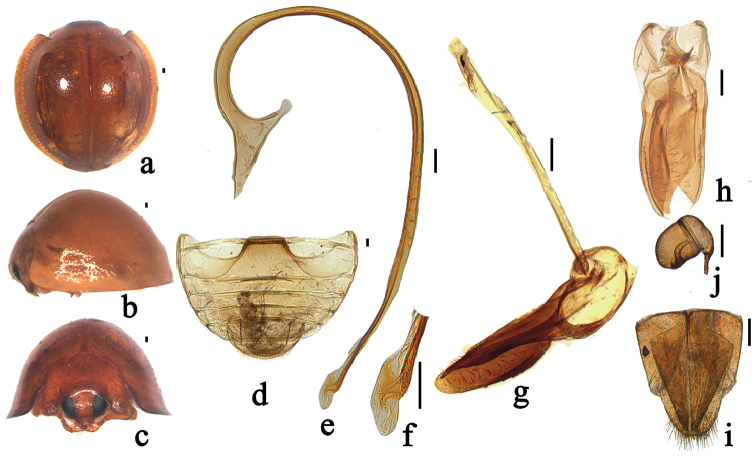
*Phaenochilus metasternalis* Miyatake, 1970. **a** dorsal view **b** lateral view **c** frontal view **d** abdomen, male **e** penis **f** apex of penis **g** tegmen, lateral view **h** tegmen, ventral view **i** ovipositor **j** spermatheca. Scale bars: 0.1 mm.

This new species can be distinguished from *Phaenochilus metasternalis* by the following combination of characters: lateral margin of elytra yellowish white (Fig. 1a–b); penis guide nearly symmetrical in ventral view, parameres slightly shorter than penis guide (Fig. 2b–c). In *Phaenochilus metasternalis*, lateral margin of elytral yellow or yellowish brown (Fig. 3a–b); penis guide distinctly asymmetrical in ventral view, slightly shorter than parameres (Fig. 3g–h).

#### Description.

TL: 3.67–3.80 mm, TW: 3.60–3.67 mm, TH: 1.87–2.07 mm, TL/TW: 1.02–1.04, PL/PW: 0.66–0.67, EL/EW: 0.96–1.00.

*Body* roundish, strongly convex (Fig. 1b). Head yellow, sparsely covered with short, greyish pubescence, eyes normally black (Fig. 1c). Pronotum yellow, only anterior angles sparsely covered with short, greyish pubescence. Scutellum and elytra yellow, lateral margin of elytra yellowish white, obvious boundary between two colors (Fig. 1a). Underside entirely yellow, except apex of mandible black with short, greyish pubescence.

*Head* relatively small, 0.44 times pronotal width, punctures on frons large, 3.0–4.0 diameters apart, surface polished between punctures; eyes subtriangular, densely faceted, widest interocular distance 0.42 times head width (Fig. 1c). Antennae composed of eight antennomeres, scape and pedicel slightly elongate, scape and pedicel of similar length and width, antennomeres 3–5 equal in length, antennomeres 6–8 gradually longer (Fig. 1e). Outer margin of mandible slightly curved (Fig. 1f). Terminal maxillary palpomere slender and elongate, approximately three times as long as basal width, with sides nearly parallel (Fig. 1g). Terminal labial palpomere slender and acuminate, rounded at apex (Fig. 1h). Pronotum 0.53 times elytral width, pronotal punctures fine but larger than those on head, 1.5–2.5 diameters apart, surface polished between punctures. Punctures on elytra fine, similar to those on pronotum, 2.0–4.0 diameters apart. Epipleuron without fovea to reccept mid and hind legs. Prosternal process short, narrow at base, gradually broadened to apex (Fig. 1d). Abdominal postcoxal lines incomplete, reaching posterior margin of abdominal ventrite 1 and running along posterior margin, almost reaching lateral margin. Posterior margin of male abdominal ventrite 5 truncate and ventrite 6 distinctly emarginate medially (Fig. 2a).

*Male genitalia*: penis slender and long, penis capsule with short outer arm and long inner arm, apex of penis with small protuberance and membranous appendage (Fig. 2d–e). Tegmen stout, penis guide gradually broadened to basal 2/5, subparallel to apical 1/5 thereafter, then gradually converging apically to blunt tip in ventral view; only one lateral margin slightly emarginate at basal 3/5 (Fig. 2c). Parameres slightly shorter than penis guide with dense, long setae at inner sides and apices with group of long setae in lateral view (Fig. 2b).

*Female genitalia*: coxites elongate, triangular (Fig. 2f). Spermatheca oblong-oval, stout, appendage of cornu well-developed (Fig. 2g).

#### Types.

Holotype, male, CHINA: Yunnan Prov: Tongbiguan, Husa, No. SCAU (E) 15235, [24°37.03'N; 97°39.05'E], *ca.* 1410m, 23.ix.2006, Wang XM leg. Paratypes. 1 male and 4 females with same data as holotype; 1 male, Yunnan Prov: Nanjingli, Ruili, [24°02.54'N; 97°52.10'E], *ca.* 811m, 25.ix.2006, Wang XM leg.

#### Distribution.

**Figure 4.**
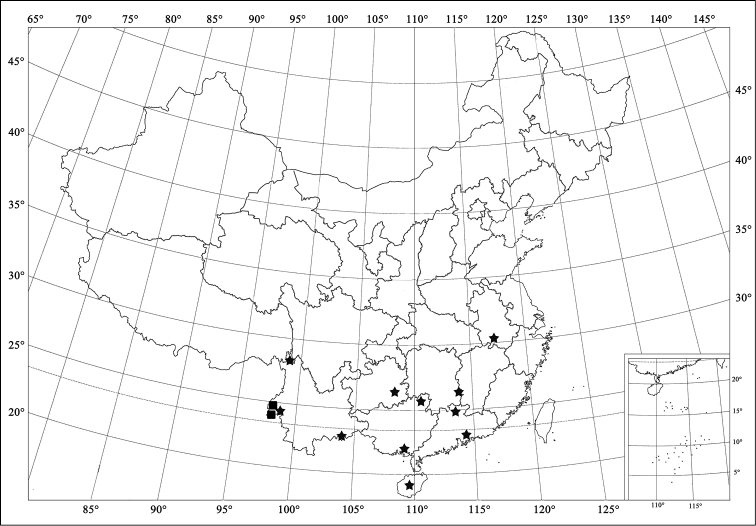
Distribution map. (■) *Phaenochilus albomarginalis* sp. n.; (★) *Phaenochilus metasternalis* Miyatake, 1970.

China (Yunnan) (Fig. 4).

#### Etymology.

The species name is derived from Latin and refers to the yellowish white lateral margin of elytra.

### Phaenochilus metasternalis

Taxon classificationAnimaliaColeopteraCoccinellidae

Miyatake, 1970

Figs 3a–j , 4

Phaenochilus metasternalis Miyatake, 1970: 334; Pang and Mao 1979: 78; Cao 1992: 152; Ren et al. 2009: 138.

#### Diagnosis.

This species can be distinguished from the other species of *Phaenochilus* by the following combination of characters: the elytral bead yellow or yellowish brown (Fig. 3a–b); male abdomen ventrite 6 weakly emarginate at middle (Fig. 3d); penis guide distinctly asymmetrical in ventral view and coxites elongate, triangular (Fig. 3h–i).

#### Description.

TL: 3.33–3.67 mm, TW: 3.13–3.47 mm, TH: 1.67–2.00 mm, TL/TW: 1.04–1.06, PL/PW: 0.72–0.74, EL/EW: 1.00–1.02.

*Body* roundish, strongly convex (Fig. 3b). Head yellow, sparsely covered with short, greyish pubescence, eyes normally black (Fig. 3c). Pronotum yellowish brown, only anterior angles sparsely covered with short, greyish pubescence. Scutellum, elytra, and elytral bead yellowish brown (Fig. 3a). Underside entirely yellowish brown or brown, except apex of mandible black with short, greyish pubescence.

*Head* relatively small, 0.56 times pronotal width, punctures on frons large, 2.5–4.0 diameters apart, surface polished between punctures; eyes subtriangular, densely faceted, widest interocular distance 0.40 times head width (Fig. 3c). Antennae composed of eight antennomeres, scape and pedicel slightly elongate, scape and pedicel of similar length and width, antennomeres 3–5 equal in length, antennomeres 6–8 gradually longer. Outer margin of mandible slightly curved. Terminal maxillary palpomere slender and elongate, approximately three times as long as basal width, with sides nearly parallel. Terminal labial palpomere slender and acuminate, rounded at apex. Pronotum 0.52 times elytral width, pronotal punctures fine but larger than those on head, 2.0–3.0 diameters apart, surface polished between punctures. Punctures on elytra fine, similar to those on pronotum, 2.0–4.0 diameters apart. Epipleuron without fovea to reccept mid and hind legs. Prosternal process short, narrow at base, gradually broadened to apex (Fig. 3d). Abdominal postcoxal lines ncomplete, reaching posterior margin of abdominal ventrite 1 and running along posterior margin, almost reaching lateral margin. Posterior margin of male abdominal ventrite 5 truncate and ventrite 6 slightly emarginte medially (Fig. 3d).

*Male genitalia*: penis slender and long, outer arm of penis capsule slightly longer than inner arm, apex of penis with a small protuberances and membranous appendage inside (Fig. 3e–f). Tegmen stout, penis guide knife-like in ventral view (Fig. 3h). Parameres as long as penis guide with dense short setae at inner sides and apices with patches of short setae visible in lateral view (Fig. 3g).

*Female genitalia*: coxites elongate, triangular (Fig. 3i). Spermatheca oblong-oval, stout, appendage of cornu well-developed (Fig. 3j).

#### Material examined.

Yunnan: 2 males, Ruili, [24°01.03'N; 97°46.23'E], *ca.* 1159m, 27.vii.2005, Wang XM leg; 1 male, Maku, Dulongjiang, [27°40.57'N; 98°18.19'E], *ca.* 1600m, 1.viii.2010, Wang XM leg; 2 females, Galaxi, Lianhuatan, Hekou, [22°57.03'N; 103°28.54'E], *ca.* 800m, 21.V.2009, Ren SX leg. Guangdong Prov: 3 males, Huangdong, Shimentai, [24°25.30'N; 113°18.28'E], *ca.* 480m, 31.x.2004, Wang XM leg;1 male, Nankunshan, Huizhou, [23°38.08'N; 113°53.34'E], *ca.* 491m, x.2004, Wang XM leg. Guangxi Prov: 1 male, Daxiagu, Maoershan, [25°50.46'N; 110°29.14'E], *ca.* 406m, 19.x.2004, Wang XM leg; 1 male, Hongqilinchang, Shiwandashan, [21°54.07'N; 107°54.26'E], *ca.* 438m, 11.xi.2004, Wang XM leg. Anhui Prov: 2 males, Huangshan, [38°08.52'N; 118°07.58'E], *ca.* 1250m, 30.vii.2005, Qin ZQ leg; 2 males, Huangshan, [38°08.39'N; 118°08.45'E], *ca.* 1367m, 14–15.ix.2010, Wang XM leg. Hainan Prov: 1 female, Yinggeling, [19°10.26'N; 109°41.08'E], *ca.* 850m, 23.xi.1997, Peng ZQ leg; 1 male, Bawangling, [19°03.51'N; 109°11.47'E], *ca.* 738m, 5.v.2005, Peng ZQ leg. Hunan Prov: 2 males, Shennonggu, Yanling, [26°30.01'N; 114°00.27'E], *ca.* 1100m, 7.x.2010, Wang XM leg. Guizhou Prov: 1 male, Datangwan, Leigongshan, [26°21.28'N; 108°10.01'E], *ca.* 1100m, 5.x.2008, Wang XM leg.

#### Distribution.

China (Anhui, Hunan, Guangdong, Guangxi, Hainan, Guizhou, Yunnan) (Fig. 4); Laos; Vietnam; Singapore; Indonesia.

## Supplementary Material

XML Treatment for Phaenochilus

XML Treatment for Phaenochilus albomarginalis

XML Treatment for Phaenochilus metasternalis
